# Introducing the Affinity Binder Knockdown Initiative⿿A public⿿private partnership for validation of affinity reagents

**DOI:** 10.1016/j.euprot.2016.01.002

**Published:** 2016-01-07

**Authors:** Tove Alm, Emma Lundberg, Mathias Uhlén

**Affiliations:** Royal Institute of Technology (KTH), Science for Life Laboratory, Box 1031, SE-17121 Solna, Sweden

**Keywords:** Antibody quality, Validation, Reproducibility, Crowdsourcing, siRNA, CRISPR

## Abstract

The newly launched Affinity Binder Knockdown Initiative encourages antibody suppliers and users to join this public⿿private partnership, which uses crowdsourcing to collect characterization data on antibodies. Researchers are asked to share validation data from experiments where gene-editing techniques (such as siRNA or CRISPR) have been used to verify antibody binding. The initiative is launched under the aegis of Antibodypedia, a database designed to allow comparisons and scoring of publicly available antibodies towards human protein targets. What is known about an antibody is the foundation of the scoring and ranking system in Antibodypedia.

## Highlights

⿢ A public⿿private partnership.
⿢ Genetic methods for antibody validation.
⿢ Crowdsourcing for improved antibody characterization.

## Graphical abstract

**Figure:**
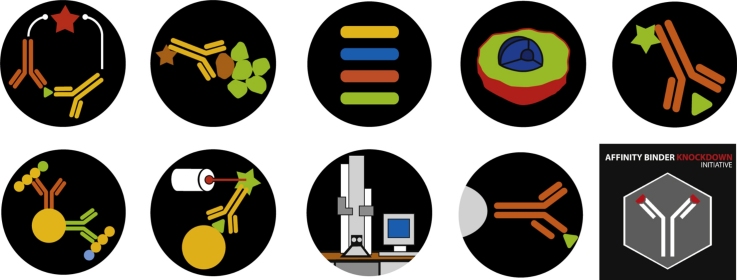

There is a large amount of publicly available affinity binders and many of them have been shown to not yield reliable results in specific applications as outlined in many articles during the last years [1], [2], [3], [4]. Several studies suggest that a large fraction of the commercially available antibodies are not functional in certain applications [5], [6]. Recently, this has been identified as a major problem in life science research, and a number of suggestions have been put forward on how to improve the quality and reproducibility of research results using antibodies [7], [8], [9], [10]. Clearly, a quality assurance program in life science research that improves the validation of antibodies for specific applications will help to save material, time, and financial resources for the user community.

Many researchers rely on published data and examine primary data, such as validation images, to make educated choices when selecting an antibody. Considering the amount of money spent on research antibodies, estimated to be $2 billion in 2014 [11], the amount of antibody related knowledge among researchers should be enormous. Many antibody providers are aware of the gold mine of knowledge residing in the research community, and currently a number of initiatives have been launched to collect data from antibody users, such as the Novus Rewards Program and the LSBio Rewards Program. These programs are tied to the manufacturers⿿ antibody portfolio, and therefore an initiative allowing a more general and broad collection of data would be of interest.

Antibodypedia (www.antibodypedia.org) is an open source database for searching and comparing validated antibodies available to the public through various providers. What is known about an antibody is the basis for the scoring and ranking system in Antibodypedia. Validation recommendation weights are applied for each application, and reference citation weights are applied to the antibody as a whole, to produce a score indicating an antibody⿿s overall performance (see Table 1). The validation and reference scores are added to a final overall score for the antibody. The score then determine the order in which both provider and antibody are listed for any gene.

**Table 1:** Validation data and citations weights.

Primary data	Score	References	Score
Supportive **knockdown**/**knockout** data in Antibodypedia (image and details present)	6	>50 citations (per antibody)	6
Supportive data in Antibodypedia (image and details present, per application)	1	21⿿50 citations (per antibody)	5
Supportive data on provider website (per application)	0.75	11⿿20 citations (per antibody)	4
Data presented in Antibodypedia (inconclusive; per application)	0.5	4⿿10 citations (per antibody)	3
Recommended by provider, but no data available (per application)	0.25	2⿿3 citations (per antibody)	2
		1 citation (per antibody)	1
[No information]	0	[No references]	0

The recently developed gene-editing techniques, using siRNA and CRISPR, offer possibilities to characterize antibodies in ways previously not possible. By using gene silencing, the expression of a target protein can be reduced or eliminated, and the antibody⿿s binding to a specific target can thus be verified using a genetic-based method. The reduced or eliminated protein expression is confirmed and visualized using any antibody based application, such as immunocytochemistry or western blot. Once the target binding of the antibody has been confirmed, the target-validation of the antibody will not need to be reconfirmed using additional methods for visualization. The importance of confirming the antibody⿿s binding to the intended target is emphasized by the size of the score given to this type of validation (see Table 1). The situation is slightly different for application specific validation of the antibody, where the aim is to assess the antibody⿿s ability to bind the target protein in a certain experimental setup. Functionality of the antibody is dependent on the context and application used, and hence validation of the antibody performance in each application is needed. Therefore, the score from the individual application specific validations are additive. Currently, Antibodypedia supports the following 15 applications: chromatin immunoprecipitation, functional assay, gel shift, immunoelectron microscopy, proximity ligation assay, radioimmunoassay, reverse phase protein array, blocking/neutralizing, ELISA, flow cytometry, immunocytochemistry, immunohistochemistry, immunoprecipitation, protein array, and western blot.

The Affinity Binder Knockdown Initiative (www.antibodypedia.com/text/knockdown_initiative) has been launched under the aegis of Antibodypedia with the aim to promote validation of antibodies using the genetic methods outlined above (siRNA and CRISPR). The initiative uses crowdsourcing to gather information from knockdown or knockout experiments performed by the research community in order to validate specific antibodies. Antibodypedia supports the initiative by offering a standardized system for sharing validation data and references about publicly available antibodies with the purpose of providing the research community with information on the effectiveness of specific antibodies in specific applications. By encouraging the research community and life science companies to collaborate, the initiative hopes to improve the knowledge about specific affinity reagents. Academic institutions and commercial companies are invited to upload information on antibody and antigen, and users of the database may submit their data to complement the existing knowledge about an antibody.

Scientists, that use knockdown or knockout validation in their research, are encouraged to upload their knockdown or knockout validation results to the publicly available database Antibodypedia. The submission process is a simple web format, and asks for information on the reagents used, experimental data (image and details on results), and a protocol (pdf); the submission process should only take a couple of minutes. Participating industrial partners include their antibodies and/or gene silencing reagents (siRNA/CRISPR) in the initiative, and will reward the first positive knockdown validation for ⿿each included reagent (antibody, siRNA, CRISPR) that is submitted to Antibodypedia on www.antibodypedia.com/validate.php. To qualify for a reward, the knockdown on protein level must be at least 50%. An example is visualized in the knockdown validation performed in Fig. 1. In this example, the staining intensity in the fluorescent images has been quantified using image analysis. Approximately 200 cells per siRNA and control, respectively, were segmented and the intensity measured. In this way an exact measure of the downregulation can be obtained as described in Stadler et al. [4]. This method confirms the antibody⿿s binding to its intended target. However, in cases where the expression is only partly silenced it is difficult to address the level of cross-reactivity.

**Fig. 1:**
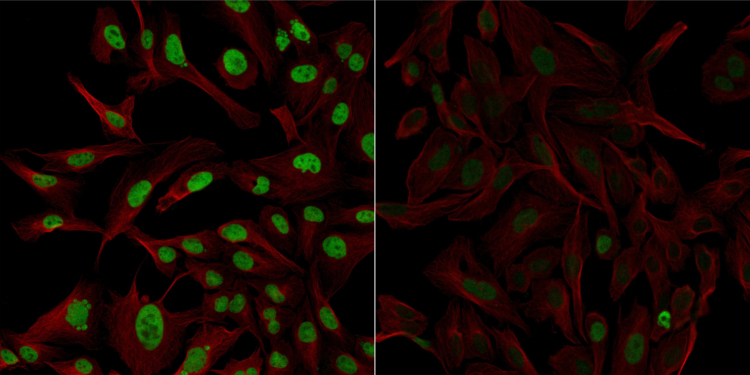
Heterogeneous nuclear ribonucleoprotein U-like 1 (HNRNPUL1) is a nuclear RNA-binding protein that binds specifically to the adenovirus E1B⿿55 kDa oncoprotein. Confocal images of immunofluorescently stained human U-2 OS cells; the protein HNRNPUL1 is shown in green and the microtubules in red. The image to the left shows cells transfected with control siRNA, and the image to the right shows cells where HNRNPUL1 has been downregulated more than 75% with specific siRNA. This data can be viewed online on www.antibodypedia.com/gene/17170/HNRNPUL1/antibody/693797/HPA046290*.*

Several antibody companies and reagent suppliers have expressed great interest in the initiative, and currently Atlas Antibodies, Aviva Systems Biology, Novus Biologicals, R&D Systems, and Invitrogen Antibodies ⿿from Thermo Fisher Scientific have included their antibodies in the Affinity Binder Knockdown Initiative. In addition, siRNA reagents from Thermo Fisher Scientific are also included in the reward program.

Identifying good affinity reagents is key to improved reproducibility and advancement of science. Most research is in one way or another relying on other scientific findings. Apart from characterizing an antibody and confirming its target binding, reproducibility is also dependent on the ability to identify the reagent used. Proper citation of antibodies in publications is important and minimum requirement is antibody provider name and catalog number. However, in a study by Vasilevsky et al., less than 50% of the antibodies were identifiable [12]. This issue has been addressed by the Resource Identification Initiative, which gives unique identifiers to antibodies. The Research Resource Identifiers (RRIDs) are free to generate, consistent across journals and papers, and machine readable [13]. The ability to easily find out in which experiments an antibody has been used in is very useful for a scientist, and the Affinity Binder Knockdown Initiative will work on providing knockdown and knockout validated antibodies with a unique identifier.

With the Affinity Binder Knockdown Initiative we hope to raise the awareness of the issues concerning the validations of antibodies and to highlight the new possibilities with the recently introduced gene-editing techniques. Ideally, providers should validate all antibodies in their portfolio using gene editing, an antibody-independent method, or with independent antibodies to confirm antibody target. This is, however, a continuous work that will not happen overnight, and in the meantime we should explore any given possibility to improve the antibody market. Many researchers have existing knowledge on individual antibodies that would be of great value to other researchers. We ask you to contribute with your knowledge on the antibodies that you use, to help make the antibody market a better place.

The Affinity Binder Knockdown Initiative started on September 28, 2015, and ends on September 30, 2016. During this period, the Affinity Binder Knockdown Initiative will be open for rewards in Europe and North America. A report of the initiative will be given at the HUPO World Conference 2016 in Taipei. If antibody providers are interested in continuing their participation, the initiative will continue in its current setup. The possibility to upload knockdown data will continue to be available on Antibodypedia despite the involvement from companies; however, the reward system is only applicable as long as companies are participating. Hopefully this initiative will serve as a starting point to unite the research community to shared efforts. One could for example envision repositories with knockdown cell lysates to allow reproducible assay testing.

Initiative organizers are Prof. Mathias Uhlén, Asst. Prof. Emma Lundberg, and Dr. Tove Alm. For specific details and submission go to: www.antibodypedia.com/text/knockdown_initiative.

## Conflict of interest

None.
